# Synthesis of nanodiamond derivatives carrying amino functions and quantification by a modified Kaiser test

**DOI:** 10.3762/bjoc.10.288

**Published:** 2014-11-20

**Authors:** Gerald Jarre, Steffen Heyer, Elisabeth Memmel, Thomas Meinhardt, Anke Krueger

**Affiliations:** 1 Institute for Organic Chemistry, Julius-Maximilians-Universität Würzburg, Am Hubland, 97074 Würzburg, Germany; 2 Wilhelm Conrad Röntgen Research Center for Complex Material Systems (RCCM), Julius-Maximilians-Universität Würzburg, Germany

**Keywords:** amino groups, carbon nanomaterials, Diels–Alder reaction, Kaiser test, nanodiamond, pyrazine

## Abstract

Nanodiamonds functionalized with different organic moieties carrying terminal amino groups have been synthesized. These include conjugates generated by Diels–Alder reactions of *ortho*-quinodimethanes formed in situ from pyrazine and 5,6-dihydrocyclobuta[*d*]pyrimidine derivatives. For the quantification of primary amino groups a modified photometric assay based on the Kaiser test has been developed and validated for different types of aminated nanodiamond. The results correspond well to values obtained by thermogravimetry. The method represents an alternative wet-chemical quantification method in cases where other techniques like elemental analysis fail due to unfavourable combustion behaviour of the analyte or other impediments.

## Introduction

Surface bound amino groups are most versatile for the grafting of larger moieties onto the surface of nanoparticles. Typically, they are used for the formation of amides using protocols from peptide chemistry or in reductive aminations [1–2]. Additionally, amino groups have an influence on the surface polarity and dispersion behaviour due to their protic character and the possibility to protonate the nitrogen.

In the case of diamond the direct amination of the surface has rarely been reported. Sotowa et al. used the photochemical reaction of ammonia with chlorinated diamond [3]. However, the termination with amino groups is typically not complete and the stability of amino groups directly bound to the diamond surface is limited as they are at least partially replaced by hydroxy groups under ambient conditions [4].

Therefore, in most cases linkers are used in order to establish stable bonding situations on the surface. These include siloxanes (e.g., APTMS) [5], alkyl chains connected to the diamond surface by amide or ester bonds [6], and aminomethyl groups formed by the nucleophilic substitution of tosylated OH groups by cyanide followed by a reduction with LiAlH_4_ [7]. Except for the last case, the linkers are coupled to the diamond surface via heteroatoms. These sites are more easily cleaved compared to carbon–carbon single bonds, e.g., in a physiological environment or other aqueous conditions. It is hence of interest to develop methods that form stable bonds between the diamond surface and the aminated moiety. One commonly used method is the (in situ) formation of aromatic diazonium salts and the reaction of the aryl residue with π-bonds, hydrogenated sites or various surface groups (in this case via heteroatoms) on the diamond surface [8–10]. Recently, we have reported an efficient method for the grafting of organic moieties onto diamond carrying sp^2^ carbon atoms at its surface using the Diels–Alder reaction of *ortho*-quinodimethanes onto π-bonds on the particle surface. By this reaction two C–C single bonds are formed in one step, thus increasing the stability of the conjugate [11]. We were able to show that the aromatic molecules immobilized by this technique can be further modified using conventional synthetic chemistry [12]. This enables the utilization of such diamond conjugates for biomedical applications as the C–C bound linker between the nanoparticle and the functional moiety is not prone to hydrolytic or enzymatic decay. Stable conjugation is an essential prerequisite for applications such as labelling or targeting.

Here we report on the grafting of nitrogen-containing heterocyclic aromatic compounds using the Diels–Alder reaction of suitable starting materials and diamond carrying sufficient amounts of sp^2^ carbon in order to allow the cycloaddition to occur. The aim was to increase the surface loading with aromatic moieties compared to the benzenic moieties due to the modified reactivity of the respective precursors.

## Results and Discussion

### The functionalization of nanodiamond with aromatic moieties carrying amino groups

**Scheme 1:**
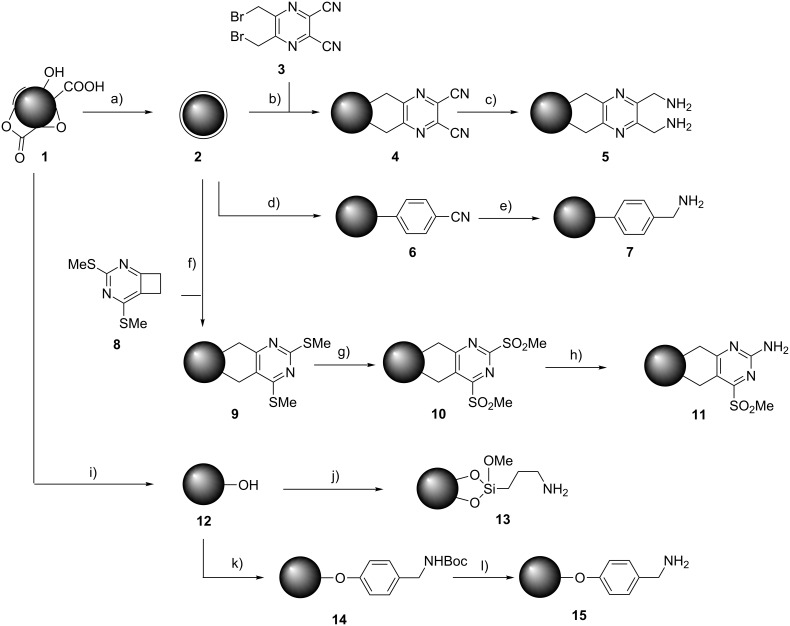
Synthesis of nanodiamond derivatives carrying primary amino groups. a) Δ, b) 18-crown-6, KI, c) BH_3_·THF, d) 4-aminobenzonitrile, isopentyl nitrite, e) BH_3_·THF, f) Δ, 1,2-dichlorobenzene, g) MCPBA, h) NH_3_, i) BH_3_·THF, j) APTMS, k) 4-aminobenzyl(*N*-Boc)amine, isopentyl nitrite, l) TFA.

For the synthesis of a broad variety of aminated nanodiamond (ND) derivatives, a series of nanodiamond conjugates with different linker systems was synthesized starting from detonation nanodiamond **1** and thermally annealed nanodiamond **2** as depicted in Scheme 1. Besides the novel functionalization methods using pyrazines and cyclobutenes (see below) several nanodiamond conjugates were prepared for comparison similar to already reported procedures.

**Figure 1:**
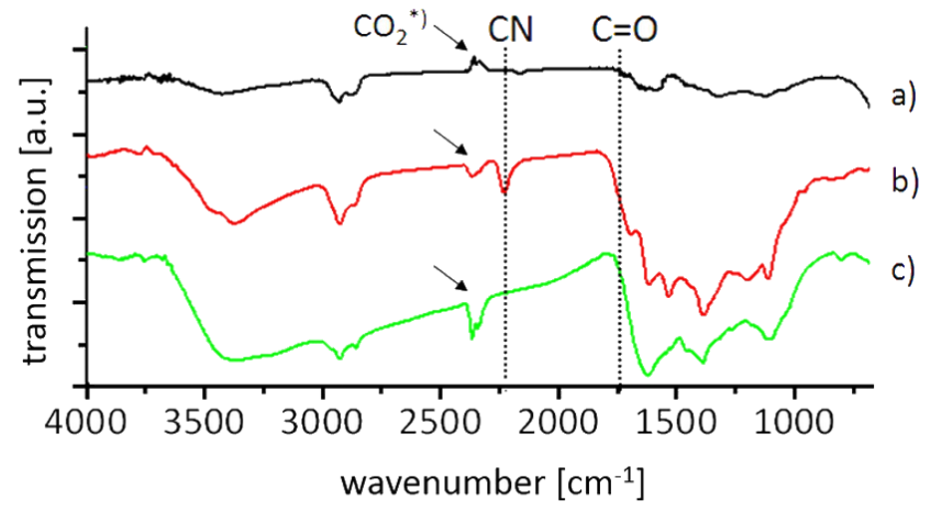
FTIR-spectra of annealed nanodiamond **2** (a), nitrile **4** (b) and amine **5** (c). As can be seen from the disappearance of the CN signal after the reduction of **4** the nitrile is fully converted into the aminomethyl group. The C=O signal position is given as reference to show the absence of respective groups. The peaks marked with an arrow (CO_2_*) at 2200–2300 cm^−1^ are due to carbon dioxide from ambient air.

It is known that pyrazine derivatives react with fullerenes in a cycloaddition reaction [13]. Pyrazine **3** was synthesized from 1,4-dibromobuta-2,3-dione and diaminomaleonitrile similar to a procedure reported by Fukunishi and coworkers [14]. The resulting 2,3-bis(bromomethyl)-5,6-dicyanopyrazine (**3**) is a precursor for the respective *ortho*-quinodimethane required for the Diels–Alder reaction. The reaction proceeds in analogy to the one for benzenic starting materials by in situ generation of the diene and reaction with surface π-bonds on the nanodiamond acting as dienophiles. The successful grafting can be monitored by the appearance of the distinct signal of nitriles in the IR spectrum at ~2225 cm^−1^ (Figure 1). In addition the aromatic vibration modes of C=N and C=C bonds are observed at 1613 and 1530 cm^−1^. The removal of solely adsorbed reagent is ensured by a thorough washing procedure and control of the washing solutions using TLC. The functionalized nanodiamond **4** forms very stable colloidal solutions in organic solvents such as acetone or dichloromethane with very low particle sizes close to the size of the pristine primary particles. The determination of the surface loading with the pyrazine moieties, however, leads to contradictory results at first sight. In the elemental analysis by combustion a surprisingly high surface loading of 1.56 mmol g^−1^ is measured whereas the thermogravimetric analysis yields a value of 0.61 mmol g^−1^. It appears that nanodiamond-carrying nitrogen-rich aromatic moieties have a different combustion behaviour compared to other diamond derivatives. The observed incomplete combustion leads to an underestimation of the carbon content and hence overestimated values for nitrogen and hydrogen (and sulfur, if present). As will be discussed below, the wet-chemical quantification supports the value obtained by thermogravimetry and hence one has to assume that combustion analysis is not suitable for this type of nanodiamond derivatives.

The dicyanopyrazine conjugate **4** can be submitted to a reduction of the nitrile groups using borane solution in THF. This mild yet efficient reaction leads to the complete transformation of the nitriles to amino groups as evidenced by the disappearance of the IR signals of the CN group. Furthermore, no difficult to remove side products are formed. The borate generated by the hydrolysis is easily washed away with water. Again, the value for the surface loading shows a strong discrepancy between TGA and elemental analysis. Both methods show the completely retained surface architecture by comparable values for the dinitrile **4** and the reduction product **5**. However, the incomplete combustion of the nanodiamond samples leads to overestimated values of 1.75 mmol g^−1^ in the case of combustion analysis. The loading determined by TGA (0.60 mmol g^−1^) is corroborated by the value obtained in the modified Kaiser test (see below). The reduction of the nitrile groups has an important influence on the colloidal stability of the ND conjugate. Whereas the nitrile derivate **4** is soluble in rather nonpolar organic solvents, the amino derivate **5** forms stable aqueous colloids with well dispersed primary particles of nanodiamond.

The use of the pyrazine-derived reagents enables the grafting of ~0.6 mmol g^−1^ of the aromatic moieties onto the particle surface, possessing 1.2 mmol g^−1^ of amino groups. Comparing this to the typical value of 0.15 mmol g^−1^ for the Diels–Alder reaction of the non-functionalized *ortho*-quinodimethane [11] proves that a significantly increased grafting is observed using the pyrazine derivative.

So far, the reported cycloaddition reactions of *ortho*-quinodimethanes leading to the grafting of aromatic rings by Diels–Alder reaction were based on the 1,4-elimination from suitable precursors. This step requires reagents such as potassium iodide and 18-crown-6 and hence the removal of the resulting side products [11]. Another approach to *ortho*-quinodimethanes makes use of the thermal ring opening of cyclobutenes. This system has been successfully used for the functionalization of fullerenes [15]. The precursor **8** was synthesized according to Martinez et al. starting from cyclobutanone and methyl thiocyanate [16]. The cyclobutene ring opens at temperatures around 190 °C and the *ortho*-quinodimethane is formed in situ. The reaction with the nanodiamond surface occurs simultaneously as soon as the diene is formed. The solvent dichlorobenzene was chosen not only for its suitable boiling point but also for the efficient solubilization of the reaction participants.

**Figure 2:**
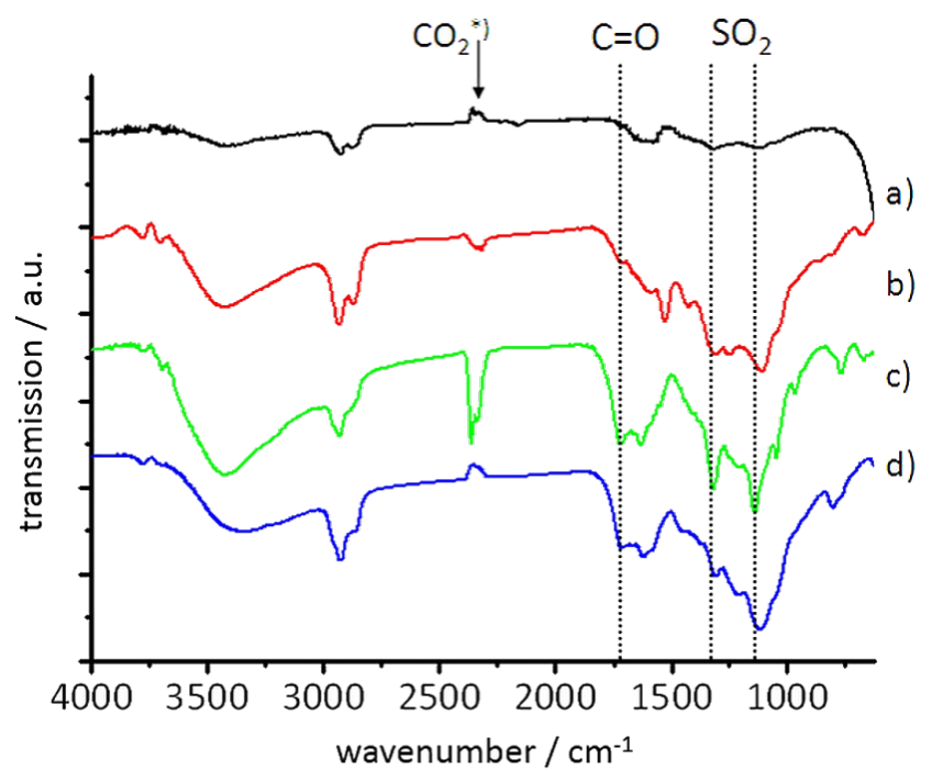
FTIR spectra (left) of compounds **2** (a), **9** (b), **10** (c) and **11** (d). The formation of the sulfone groups in **10** and the reduction of signal intensity for **11** are clearly visible. In the spectrum of **10** and **11** the carbonyl signals generated by the side reaction of MCPBA with surface groups are marked with a dotted line (the CO_2_ signal is due to ambient air).

The ND conjugate **9** shows characteristic signals in the IR spectrum (Figure 2), namely the vibrations at 1590, 1530 and 1430 cm^−1^ are caused by the grafted heteroaromatic moieties. The spectrum corresponds well to the signals observed for 2,4-bis(methylthio)pyrimidine [17]. The surface loading was determined by TGA and elemental analysis based on the nitrogen and sulfur content. All analytical methods yield comparable results around 0.2 mmol g^−1^.

The further reaction depicted in Scheme 1 enables the formation of an aromatic amine at the 2-position of the ring by the oxidation of the thioethers and subsequent substitution of the sulfone by an amino function. Throughout the transformation the surface loading (see TGA data and elemental analysis in the experimental part) with the organic moieties remains the same showing the high stability of the double grafting by covalent C–C bonds. However, during the oxidation of the thioethers to the sulfones a side reaction has been observed. MCPBA reacts not only with the sulfur-containing groups in **9** but also with the annealed diamond surface itself as evidenced by the emergence of IR signals of carbonyl and ether bonds besides the expected signals at 1320 and 1140 cm^−1^ for the symmetric stretching mode of the sulfones (see spectra c) and d) in Figure 2). A control experiment using nanodiamond not functionalized with the pyrimidine proved the reactivity of surface groups of the nanodiamond (CH, OH, π-bonds etc. are present on thermally annealed nanodiamond [18]) with MCPBA showing the formation of oxidized surface groups and an increase of the respective IR signals for carbonyl groups (see Supporting Information File 1 for details). When the mass loss related to the groups generated by this side reaction is subtracted from the overall result using the results from the control experiment, the values for the surface loading for compound **10** measured by combustion analysis (using values for N and S and hence only considering the heterocycle) and TGA (where the combined mass loss for the heterocycle and the oxidized groups is measured) are fully comparable.

The generation of the amino functions is carried out using gaseous ammonia and hence the isolation and purification of the reaction product **11** is very simple. As expected the intensity of the sulfone related IR bands decreases upon amination (but does not vanish as only the SO_2_Me group in 2-position is exchanged) and new signals corresponding to amino functions (1590 and ~3450 cm^−1^) appear in the spectrum. Furthermore, the sulfur content of the material is reduced to one half of the original value. This shows that the reaction occurs with high efficiency as the remaining sulfur stems from the conserved sulfone group.

From these results it is obvious that the thermal ring opening of suitable cyclobutenes is another valuable addition to the portfolio of cycloaddition related surface reactions of nanodiamond. It allows the efficient immobilization of quite complex organic moieties without the use of auxiliaries or further reagents. No problematic side products or waste are formed and the selective transformation of the different functional groups on the aromatic ring also allows the orthogonal functionalization with different moieties in the subsequent grafting steps.

### Analysis of surface amino groups on nanodiamond

Besides spectroscopic techniques, thermogravimetry and combustion (elemental) analysis are typically applied for the quantification of surface groups on nanomaterials. In most cases these methods yield reliable results and the obtained values are comparable for the different methods. However, it turned out that for some nanodiamond derivatives with a high concentration of nitrogen in the surface functionalities the discrepancy between surface loadings determined using elemental analysis and TGA was exceeding the variations brought upon by different measurement methods and experimental deviations. As shown in Table 1 the values obtained by combustion analysis were three times higher than those measured by TGA. Reasons for this difference can be the incomplete combustion and soot formation during elemental analysis. As evidenced by black residues in the crucibles used for the combustion this hypothesis is reasonable. Another aspect to be taken into consideration is the accessibility of functional groups. Using elemental analysis the entire amount of nitrogen in the sample will be determined, including nitrogen in the diamond lattice (which can be segregated of nitrogen content in the pristine material after the measurement) [19] and all amino groups anywhere in the sample. However, this does not mean that all these amino groups are accessible, e.g., for further coupling reactions due to agglomeration or porosity of the material.

**Table 1:** Surface loading of nanodiamond-carrying amino groups on different linkers using three independent quantification methods.

sample (sample mass in Kaiser test /mg)	surface loading (mmol g^−1^) Kaiser test	surface loading (mmol g^−1^) elemental analysis	surface loading (mmol g^−1^) TGA^a^

**5** (0.8)	0.60	1.75^b^	0.61
**7** (0.7)	0.17	0.22	0.19
**11** (–)	–^c^	0.23 (0.20)^d^	0.23
**13** (1.2)	1.05	1.07	0.49^e^
**15** (2.0)	0.27	0.29	0.32

^a^Surface loading has been calculated from the mass loss step related to the removal of the organic matter. The desorption of water has been ensured by heating to 120 °C where the desorption step ends, ^b^this unexpectedly high value is due to the incomplete combustion of this sample type; ^c^aromatic amines cannot be quantified using the Kaiser test as no proton in the α-position is available, the qualitative test was negative as expected; ^d^value based on sulfur (nitrogen) content; ^e^the thermogravimetric analysis of silanized samples often gives less pronounced steps in the thermogram, making the quantification less reliable, furthermore the formation of a stable silica shell reduces the observed mass loss.

Furthermore, the cleavage of strongly bound organic moieties from the surface will occur only at rather elevated temperature. In this case the thermogravimetric step will be less pronounced as it coincides with the early sublimation of carbonaceous material from the diamond surface. The latter sets in at temperatures above 450 °C, where the cleavage and desorption of strongly bound species is not yet completed. Hence, this overlay makes an unambiguous determination of the step temperatures in TGA challenging. It is therefore attractive to find other, independent methods for the quantitative determination of functional groups on the diamond surface, especially those imparting high nitrogen content, such as amino groups.

A very popular technique for the quantification of primary amino groups is the so-called Kaiser test [20]. It makes use of the formation of Ruhemann’s Blue by the reaction of ninhydrine with the primary amino groups [21–22]. A major application of this assay is the test on residual amino groups of amino acids in solid-phase supported peptide synthesis. A report on a quantitative variation of the Kaiser test was published by Sarin et al. [23]. As this assay is colorimetric, its execution is very convenient. One limitation of the method, though, is the restriction to primary amines. Secondary amines cannot be detected reliably and aromatic and tertiary amines do not yield positive test results either [24]. One report on the non-quantitative application of the Kaiser test on ethylene diamine functionalized nanodiamond has been reported recently [25].

For comparison, the monoaminated diamond conjugates **7**, **13** and **15** have been synthesized (see Supporting Information File 1 for details). As it can be seen from the analytical data the surface loading with amino groups for **7** and **15** is about ~0.2 mmol g^−1^, a very typical value for this type of reaction. The observed surface loading for **13** is higher due to secondary reactions of already grafted alkoxy silanes [5].

**Figure 3:**
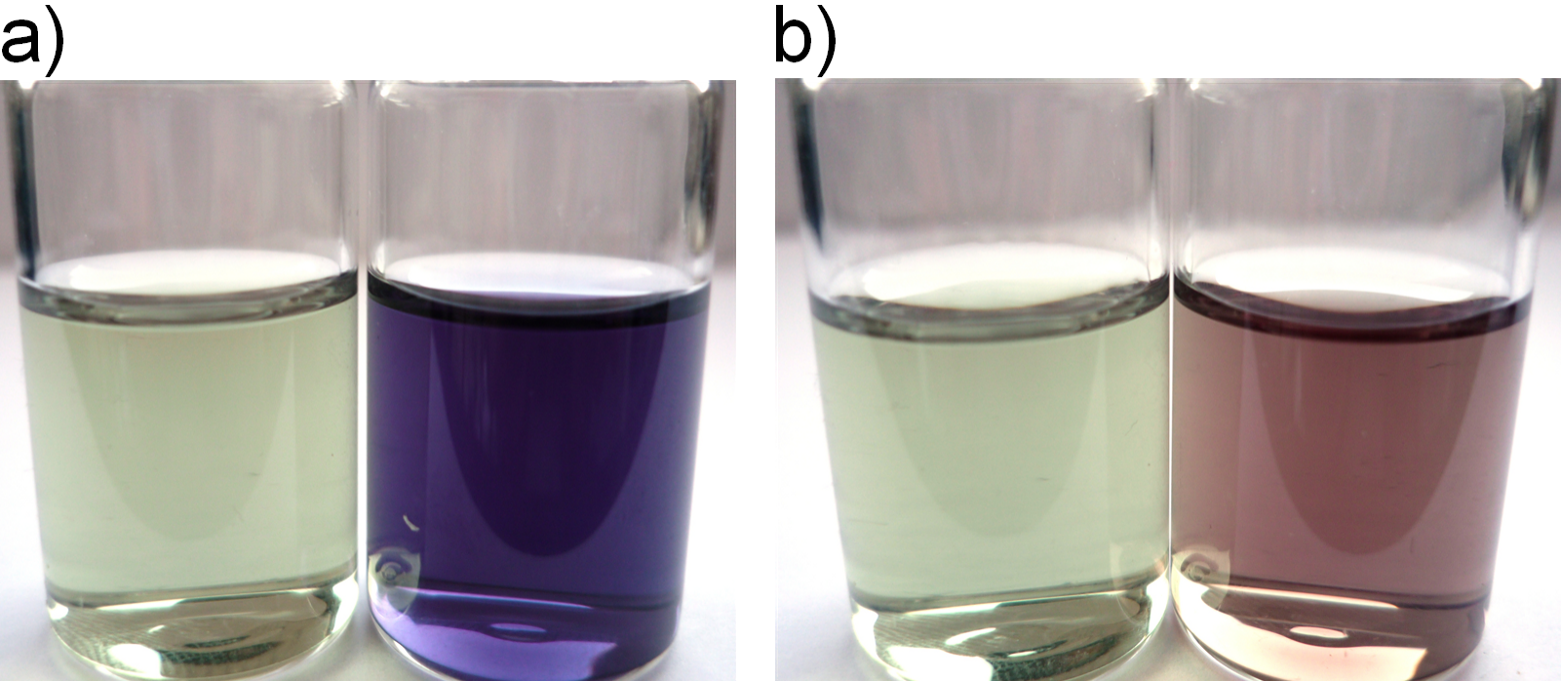
Modified Kaiser test. a) Left: control, right: positive result; b) left: control, control with premature addition of 60% ethanol solution (before solution has fully cooled down and colour has vanished).

In a first series of experiments the assay was carried out on nanodiamond samples **5** and **15** using the protocol reported by Troll et al. [22]. Surprisingly, very low values for the amino group loading were obtained for these samples in the first attempts of wet-chemical analysis (see Supporting Information File 1 for details). However, as **15** did not show a discrepancy between combustion and thermogravimetric analysis the Kaiser test was apparently yielding false results. To elucidate the origin of the deviation a deeper look into the mechanism of the formation of Ruhemann’s Blue is required. An important intermediate is hydrindantin, which is formed upon reduction using KCN (see Supporting Information File 1 for a complete reaction mechanism). Its role in the process is still under discussion, and the rate constant of the reaction depends on the hydrindantin concentration in a complex manner – emphasizing its role as an important intermediate [26–27]. Moore and coworkers reported that in the event of insufficient amounts of reducing agent the formation of the coloured species is too low and variable [28]. Although the amount of KCN in the standard protocol is taking this finding into account we suspected that nanodiamond itself could be adsorbing the intermediate species and thus hampering the further proceeding of the reaction. It is well known, that hydrophilic nanodiamond (and samples carrying amino groups count among these) is prone to unspecific adsorption of organic molecules via hydrogen bonding and other interactions [29]. Therefore it seems reasonable to assume that the rather low amounts of hydrindantin formed upon the addition of KCN can be adsorbed on the diamond surface and hence withdrawn from the reaction mixture. By forming higher amounts of hydrindantin using increased amounts of the reducing agents and thus increasing the concentration of the free intermediate in the solution we hoped to overcome this issue. By tripling the amount of KCN it was indeed possible to obtain reproducible values for the surface loading with NH_2_ groups that correspond to the values obtained by other methods. Due to the increased KCN concentration and the increased formation of the intermediate hydrindantin the reaction mixture shows a characteristic red colouring at elevated temperatures as already reported by Sarin et al. [23]. Upon cooling, this colour vanishes almost completely (only a weak residual absorption at 570 nm is observed in the UV–vis spectrum, which has to be taken into account during the quantification). It has to be mentioned that the addition of 60% ethanol solution should be executed only after the disappearance of the red colour. The back oxidation is otherwise hindered and a quantitative determination is rendered impossible (Figure 3).

In order to calibrate the measurement a reference sample for the colorimetric assay was used. Using benzylamine at different concentrations in water a standard graph was established for the new test protocol (with triple amount of KCN and delay before the addition of the ethanol) measuring the extinction of the Ruhemann’s Blue in the absorption spectra. Additionally, the required reaction time was determined using nanodiamond **5** with the same protocol and taking samples of 1 mL at time intervals. After work-up these samples were submitted to the colorimetric assay and a minimum duration until complete transformation was found to be in the range of 10 min. Using this optimized assay protocol a series of aminated nanodiamond samples have been examined (Table 1). Furthermore, the dicyano derivative **4** and the Boc-protected derivative **14** were tested and showed a negative test result as expected. The results of the Kaiser test for compounds **5**, **7**, **13** and **15** show a high reproducibility and correspond well within the error limits to the values obtained by thermogravimetry in all cases except **13** and with elemental analysis when complete combustion of the nanodiamond derivative is achieved. The deviation of the TGA results in the case of **13** is most likely due to the incomplete removal of the siloxane shell, which can crosslink and form a glassy layer around the nanodiamond core upon heating. This reduces the apparent surface loading as complete removal is assumed in the calculation. As can be seen from the results, the modified Kaiser test is a valuable tool for the quantification of amino groups on nanoparticle surfaces, namely when only low amounts of sample are available, e.g., when using fluorescent nanodiamond as the starting material or when the material shows unfavourable properties in other analytical methods. However, care should be taken in the event of strong agglomeration of the nanoparticles. Only the surface groups accessible by the reagents will be measured using this wet-chemical method.

## Conclusion

In summary, we have established two new functionalization methods for the covalent grafting of organic moieties onto nanodiamond using pyrazine and cyclobutene precursors for the in situ formation of *ortho*-quinodimethanes. These can be used as dienes in Diels–Alder reactions with π-bonds on the surface of nanodiamond. The use of pyrazine **3** results in a significantly increased grafting rate compared to other aromatic moieties linked by carbon–carbon bonds. Furthermore, a modified Kaiser test for the quantitative analysis of primary amino groups on nanodiamond has been developed and tested with a broad variety of aminated diamond samples. The modified conditions ensure the formation of sufficient amounts of the required hydrindantin intermediate and deliver reproducible and comparable results. The method can be used for other nanoparticles with similar adsorption properties as well and represents a useful addition to the wet-chemical analysis of functional groups on nanomaterials.

## Experimental

### General chemicals and methods

Detonation diamond has been purchased from Gansu Lingyun Corp. (Lanzhou, China). All other chemicals have been purchased from Aldrich and Fluka and have been used without further purification. Solvents have been dried according to standard procedures.

The following apparatus have been used for the experimental work:

Sonication: Ultrasound bath: Bandelin Sonorex Digitec Type DT52 (max. 80 W, 35 kHz), centrifuges: Hettich EBA 21 Type 1004, FTIR: Perkin-Elmer 1600 Series with ATR equipment and home-made vacuum cell (KBr pellet). Samples for measurements using the vacuum cell were heated at 100–120 °C for 2 h in vacuo in order to remove most of the water adsorbed on the diamond surface; UV–vis: Kontron Instruments Uvikon 943, Thermogravimetry (TGA): Perkin Elmer STA 6000, measurements were performed in high purity nitrogen with a heating rate of 10 °C min^−1^ (ca. 20 mg of sample were used for each analysis); elemental analysis: Euro Vector Euro EA 3000 Series; NMR: Bruker AC 250, tube furnace: Thermolyne Type 21100.

### Reagents for Kaiser test

1.) Buffer pH 5.5: 36 g of sodium acetate was dissolved in 6.9 mL of conc. acetic acid and distilled water was added until a volume of 100 mL was reached.

2.) 5% ninhydrin solution: 5 g of ninhydrin were dissolved in 100 mL of ethanol.

3.) KCN pyridine reagent: 2 mL of 0.03 M KCN solution were diluted to a volume of 100 mL with pyridine.

4.) Phenol solution: 80 g of phenol were dissolved in 20 mL of ethanol while being gently heated.

5.) Ethanol solution: 60 mL of ethanol was diluted with 40 mL of deionized water.

### Procedure for the quantification of amino groups on nanodiamond

The nanodiamond carrying amino groups (typically 0.5–2 mg of the powder) was suspended in 1 mL of dist. water. To this suspension 1 mL of the buffer solution (no. 1, see above) was added and the mixture was sonicated in an ultrasonic bath for 15 min. After that, 1 mL of the KCN solution (no. 3) and 1 mL phenol solution (no. 4) were added and the suspension was heated at 120 °C (oil bath temperature) for 10 min, 1 mL of ninhydrin solution (no. 2, see above) was added and heated for another 10 min. The solution was cooled to room temperature within 30 min and 5 mL of the ethanol solution was added. The solid was separated by centrifugation and from the supernatant a UV-spectrum was recorded.

The following equation was used for the quantification:

surface loading [mmol g^−1^] 
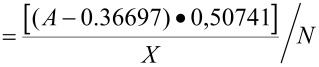
 with

*A* = absorption; *X* = weight of diamond sample [g]; *N* = number of amino groups per molecule. The absolute values are obtained from the calibration curve measured with benzylamine. For the calibration curve and the reaction time optimization see Supporting Information File 1.

### Synthesis of aminated nanodiamond samples

**Synthesis of 4:** A suspension of 250 mg of annealed detonation diamond **2** (2 h, 750 °C, vacuum), 1.16 g (6.96 mmol) potassium iodide, 520 mg (1.81 mmol) 2,3-bis(bromomethyl)-5,6-dicyanopyrazine (**3**) and 660 mg (2.50 mmol) 18-crown-6 in 10 mL of abs. toluene was heated for 72 h under reflux and nitrogen atmosphere. After cooling the diamond particles were isolated by centrifugation (15000 min^−1^, 10 min). The precipitate was washed eight times with acetone, three times with water, three times with acetone and three times with dichloromethane in consecutive dispersion/centrifugation cycles. 150 mg (60% yield) of a black solid was obtained as the product. Elemental analysis: N: 10.4%, C: 74.1%, H: 1.7%; FTIR (vacuum cell) 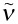: 3370, 2923 (CH), 2227 (CN), 1635, 1614, 1529, 1384, 1197, 1110. 842, 696 cm^−1^; TGA (% weight loss): 120–460 °C: 9.6% (fragment: C_8_H_4_N_4_; 156 g mol^−1^); Surface loading: 1.56 mmol g^−1^ (calculated from EA), 0.61 mmol g^−1^ (calculated from TGA), Kaiser-Test: negative, zetapotential: not measured as no stable colloidal solution in water was obtained. Particle size: 15 nm (acetone).

**Synthesis of 5:** 65 mg of **4** was suspended in 5 mL BH_3_∙THF (1 M) and heated for 20 h under nitrogen atmosphere at 50 °C. Excess borane was decomposed by adding 2 N HCl. The diamond particles were isolated by centrifugation (15000 min^−1^, 10 min). The precipitate was washed six times with a mixture of dioxane/water (9:1), three times with acetone and three times with dichloromethane in consecutive dispersion/centrifugation cycles. 49 mg (75% yield) of a black solid was obtained as the product. Elemental analysis: N: 11.2%, C: 68.4%, H: 3.9%; FTIR (vacuum cell) 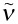: 3359, 3215, 2924 (CH), 2854 (CH), 1620 (NH), 1384, 1261, 1108, 799, 673, 614 cm^−1^; TGA (% weight loss): 140–300 °C: 10.0% (fragment: C_8_H_12_N_4_; 164 g mol^−1^); Quantitative Kaiser test: UV: A = 2.2501 (568 nm); Surface loading: 1.75 mmol g^−1^ (calculated from EA), 0.61 mmol g^−1^ (calculated from TGA), 0.60 mmol g^−1^ (Kaiser-Test); zetapotential: 19.9 mV, particle size: 13 nm (water).

**Synthesis of 9:** 120 mg of annealed nanodiamond **2** was suspended in a solution of 120 mg (0.61 mmol) of **8** in 10 mL 1,2-dichlorobenzene and heated to reflux for 20 h under nitrogen atmosphere. The solid was recovered by centrifugation and the supernatant was discarded. The solid was washed six times with acetone, five times with water and five times with dichloromethane in consecutive dispersion/centrifugation cycles. After drying at 70 °C for 24 h the product was obtained as a dark grey solid. Yield: 101 mg (84%). Elemental analysis: C: 88.9%, H: 1.3%, N: 2.9%, S: 1.4%; FTIR (vacuum cell) 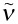: 3430 (br), 2934 (s, ν(C-H)), 2871 (s, ν(C-H)), 1589 (w, ν(C=C_arom._)), 1528 (m, ν(C=C_arom._)), 1425 (m, ν(C=C_arom._)), 1312 (w), 1252 (w), 1110 (m), 860 (w), 810 (w), 677 (m) cm^−1^; TGA (% weight loss): 150–540 °C: 4.2% (C_8_H_10_N_2_S_2_); surface loading: 0.23 mmol g^−1^ (calculated from sulfur content in EA), 0.21 mmol g^−1^ (calculated from nitrogen content in EA), 0.21 mmol g^−1^ (calculated from TGA); zetapotential: +34.2 mV (measured in aqueous dispersion).

**Synthesis of 10:** 50 mg of functionalized nanodiamond **9** were suspended in a solution of 170 mg (0.99 mmol) *meta*-chloroperoxybenzoic acid (MCPBA) in 10 mL dry dichloromethane and stirred for 20 h at room temperature under nitrogen atmosphere. The solid was separated by centrifugation and the supernatant was discarded. The solid was washed six times with dichloromethane, six times with acetone and six times with water in consecutive dispersion/centrifugation cycles. After drying at 70 °C for 24 h the product was obtained as a grey solid. Yield: 40 mg (80%). Elemental analysis: C: 85.9%, H: 1.2%, N: 3.0%, S: 1.3%; FTIR (vacuum cell) 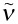: 3433 (br), 2931 (m, ν(C-H)), 1722 (m, ν(C=O)), 1630 (m), 1550 (w), 1405 (w), 1320 (s, ν(C_2_SO_2_)), 1211 (w), 1140 (s, ν(C_2_SO_2_)), 1046 (m), 968 (m), 770 (m), 670 (m) cm^−1^; TGA: (% weight loss): 130–520 °C: 7.0% (C_8_H_10_N_2_O_4_S_2_) (including 1.4% direct surface oxidation by MCPBA as demonstrated in a control experiment); surface loading: 0.21 mmol g^−1^ (calculated from sulfur content in EA), 0.23 mmol g^−1^ (calculated from nitrogen content in EA), 0.21 mmol g^−1^ (calculated from corrected TGA data); zetapotential: +19.8 mV (measured in aqueous dispersion).

**Synthesis of 11:** 20 mg of the functionalized nanodiamond **10** were suspended in 5 mL dichloromethane and stirred at room temperature while gaseous ammonia was bubbled through the solution for 6 h. The solid was separated by centrifugation and the supernatant was discarded. The solid was washed six times with acetone and six times with water in consecutive dispersion/centrifugation cycles. After drying at 70 °C for 24 h the product was obtained as grey solid. Yield: 17 mg (85%); Elemental analysis: C: 82.0%, H: 1.3%, N: 3.0%, S: 0.7%; FTIR (vacuum cell) 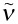: 3342 (br), 2925 (s, ν(C-H)), 2869 (m, ν(C-H)), 1716 (m, ν(C=O)), 1622 (m), 1585 (m, δ(NH)), 1312 (s, ν(C_2_SO_2_)), 1207 (w), 1120 (s, ν(C_2_SO_2_)), 800 (m) cm^−1^; TGA (% weight loss): 140–510 °C: 5.9% (including 1.4% mass loss from direct surface oxidation (see above); surface loading: 0.23 mmol g^−1^ (calculated from sulfur content in EA), 0.20 mmol g^−1^ (calculated from nitrogen content in EA), 0.23 mmol g^−1^ (calculated from corrected TGA); zetapotential: +12.2 mV (measured in aqueous dispersion).

## Supporting Information

File 1 Details for calibration of the Kaiser test, test robustness and optimization of the reaction time, reaction mechanism for colorimetric assay; synthesis of organic precursor compounds and further nanodiamond derivatives.
