# Supplementary material for: Synthesis of nanodiamond derivatives carrying amino functions and quantification by a modified Kaiser test
Source: Beilstein J Org Chem. 2014 Nov 20;10:2729–37. doi: 10.3762/bjoc.10.288 (PMC4273261; doi:10.3762/bjoc.10.288)
Supplement: File 1 — Details for calibration of the Kaiser test, test robustness and optimization of the reaction time, reaction mechanism for colorimetric assay; synthesis of organic precursor compounds and further nanodiamond derivatives. [file Beilstein_J_Org_Chem-10-2729-s001.pdf]

# Supporting Information

# for

# Synthesis of nanodiamond derivatives carrying amino functions and quantification by a modified Kaiser test

Gerald Jarre[1], Steffen Heyer[1], Elisabeth Memmel[1], Thomas Meinhardt[1] and Anke Krueger*[1,2]

Address: [1]Institute for Organic Chemistry, Julius-Maximilians-Universität Würzburg, Am Hubland, 97074 Würzburg, Germany and [2] Wilhelm Conrad Röntgen Research Center for Complex Material Systems (RCCM), Julius-Maximilians-Universität Würzburg

Email: Anke Krueger - anke.krueger@uni-wuerzburg.de

*Corresponding author

## Details for calibration of the Kaiser test, test robustness and optimization of the reaction time, reaction mechanism for colorimetric assay; synthesis of organic precursor compounds and further nanodiamond derivatives

### Synthetic procedures:

### Production of thermally annealed nanodiamond:

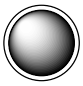

**2**

1.00 g of the pristine diamond was placed in a tube furnace and heated *in vacuo* ($10^{-4}$ mbar) to 750 °C. The heating rate was 100 °C / min and the furnace was allowed to cool to room temperature within 2 h (while maintaining the vacuum).

**FT-IR** (vacuum cell): $\tilde{\nu}$ = no specific absorption.

**Elemental analysis**: N: 2.1 %, C: 95.1 %, H: 0.7 %.

**Synthesis of hydroxylated nanodiamond 12:**

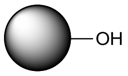


**12**

The synthesis was carried out according to the procedure reported in [1].

**Elemental analysis:** C: 90.8 %, H: 1.0 %, N: 2.3 %.
**FT-IR** (vacuum cell): $\tilde{\upsilon}$ = 3411, 2924, 1630, 1516, 1115 $cm^{-1}$.

**Synthesis of diene precursor 3:**

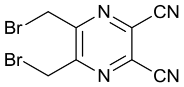


**3**

The synthesis was carried out according to the procedure reported in [2] using 2.44 g (10.0 mmol) 1,4-dibromobut-2,3-dione and 1.08 g (10.0 mmol) diaminomaleonitrile in 100 ml benzene. Yield: 1.22 g (3.87 mmol, 39 %) pale yellow solid.

The spectroscopic data correspond to the data reported in the literature.
**$^1$H-NMR** ($CDCl_3$): δ = 4.75 (s, 4H) ppm.
**Melting point**: 83 °C (recryst. from benzene, uncorrected)

**Synthesis of aminosilanized nanodiamond 13:**

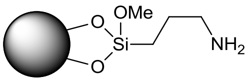


**13**

The synthesis was carried out according to the procedure reported in [1] using 50 mg of nanodiamond **12** and 10 ml of 5% 3-(aminopropyl)trimethoxysilane in dry acetone. Yield: 44 mg of a grey solid.

**Elemental analysis:** C: 80.0 %, H: 2.1 %, N: 3.6 %.
**FT-IR** (vacuum cell): $\tilde{\upsilon}$ = 3298, 2925 (CH), 2856 (CH), 1707, 1661 (NH), 1623, 1445, 1382, 1307, 1210, 1110, 804 $cm^{-1}$.
**TGA** (% weight loss): 120-580 °C: 7.2 % (fragment: $C_4H_{11}N$; 73 g $mol^{-1}$).
**Quant. Kaiser-Test**: A = 3.2620 (568 nm).
**Surface loading:** 1.07 mmol $g^{-1}$ (calculated from EA)
0.98 mmol $g^{-1}$ (calculated from TGA)
1.05 mmol $g^{-1}$ (Kaiser-Test).

**Synthesis of aminated nanodiamond 15:**

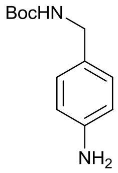


The synthesis was carried according to the procedure reported in [3] using 1.00 g (8.18 mmol) 4-aminobenzylamine, 25 ml THF/$H_2O$ (2:1) and 1.97 g (9.00 mmol) $Boc_2O$. Yield after column chromatography (silica, ethylacetate/hexane 1:2): 1.56 g (86%) of an orange solid

**$^{1}$H-NMR** ([$d_6$]-dmso): δ = 7.11 (t, 1H, $^{3}J$ = 5.4 Hz), 6.87 (d, 2H, $^{3}J$ = 8.4 Hz), 6.48 (d, 2H, $^{3}J$ = 8.4 Hz), 4.89 (s, 2H), 3.92 (d, 2H, $^{3}J$ = 6.0 Hz), 1.38 (s, 9H) ppm.
**$^{13}$C-NMR ([$d_6$]-dmso):** δ = 28.26 (C(*C*$H_3$)$_3$), 43.10 (*C*$H_2$), 77.43 (*C*($CH_3$)$_3$), 113.63 (*C*$H_{Ar}$), 127.13 (*C*$_{q,Ar}$$CH_2$), 127.94 (*C*$H_{Ar}$), 147.32 (*C*$_{q,Ar}$$NH_2$), 155.65 (*C*OO) ppm.
**FTIR (ATR):** $\tilde{\nu}$ = 3452 (w), 3367 (m), 2980 (w), 2933 (w), 1686 (s), 1625 (m), 1514 (vs), 1362 (m), 1244 (s), 1157 (s), 836 (m), 822 (m) $cm^{-1}$.
**MS (EI):** m/z = 222.1 (M+, 7.38), 165.0 (M+ - C($CH_3$)$_3$, 100.0), 121.1 (M+ - Boc, 29.8), 106.1 (M+ - NHBoc, 84.5), 57.1 (C($CH_3$)$_3$, 28.2), 44.0 ($CO_2$, 3.34).
**Melting point**: 79 - 80 °C

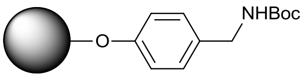


**14**

200 mg of diamond **12** was suspended in 50 ml of water and sonicated for 20 min. After heating to 80 °C 800 mg (4.00 mmol) of the protected aminobenzylamine and 586 mg (5.00 mmol) isopentyl nitrite were added and the mixture was stirred for 20 h at 80 °C. After cooling the diamond particles were isolated by centrifugation. The precipitate was washed repeatedly eight times with acetone until the supernatant was colorless and two times with dichloromethane in consecutive washing / centrifugation cycles. 181 mg of a brownish grey solid was obtained as the product.

**Elemental analysis:** C: 90.0 %, H: 1.4 %, N: 3.6 %.
**FT-IR** (vacuum cell): $\tilde{\nu}$ = 2981 (CH), 1935, 1692 (CO), 1512, 1385, 1369, 1172, 832 $cm^{-1}$.
**Particle size ($H_2O$):** 10 % < 29 nm, 50 % < 40 nm, 90 % < 75 nm.
**TGA** (% weight loss): 120 - 480 °C: 6.0 % (fragment: $C_{12}H_{16}NO_3$; 222 g $mol^{-1}$).
**Surface loading:** 1.25 mmol $g^{-1}$ (calculated from EA)
0.27 mmol $g^{-1}$ (calculated from TGA).
**Kaiser-Test**: negative

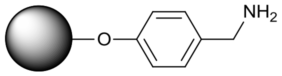


**15**

100 mg functionalized diamond **14** was sonicated for 20 min in 9 ml of dry dichloromethane, 1 ml of trifluoroacetic acid was added and the reaction mixture was stirred over night at room temperature. The diamond particles were isolated by centrifugation. The precipitate was

washed repeatedly until pH 7, three times with acetone and three times with dichloromethane in consecutive washing / centrifugation cycles. 63 mg of a brownish solid was obtained as the product.

**Elemental analysis:** C: 88.0 %, H: 1.1 %, N: 2.4 %.
**FT-IR** (vacuum cell): $\tilde{\nu}$ = 3263, 2927 (CH), 1673 (NH), 1421, 1207, 1136, 832, 802, 72 $cm^{-1}$.
**TGA** (% weight loss): 140-460 °C: 3.8 % (fragment: $C_7H_8NO$; 122 g $mol^{-1}$).
**Particle size ($H_2O$):** 10 % < 16 nm, 50 % < 21 nm, 90 % < 36 nm.
**Quant. Kaiser-Test**: A = 1.4389 (568 nm).
**Surface loading:** 0.29 mmol $g^{-1}$ (calculated from EA)
0.31 mmol $g^{-1}$ (calculated from TGA)
0.27 mmol $g^{-1}$ (Kaiser-Test)

**Synthesis of aminated nanodiamond 7:**

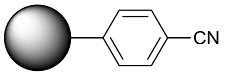


**6**

150 mg of annealed nanodiamond **2** (2 h, 750 °C, vacuum) was sonicated in 30 ml of 1 M HCl for 15 min. After heating to 80 °C 470 mg (4.00 mmol) 4-aminobenzonitrile and 530 mg (4.00 mmol) isopentyl nitrite was added. The mixture was stirred for 14 h at 80 °C. After cooling the diamond particles were isolated by centrifugation. The precipitate was washed repeatedly five times with acetone, two times with DMSO, three times with water and two times with dichloromethane in consecutive washing / centrifugation cycles. 127 mg (85 % yield) of a gray solid was obtained as the product.

**Elemental analysis:** C: 90.5 %, H: 1.2 %, N: 2.7 %.
**FT-IR** (vacuum cell): $\tilde{\nu}$ = 3256, 2929 (CH), 2882 (CH), 2228 (CN), 1741, 1655, 1605, 1500, 1375, 1317, 1228, 1154, 844 $cm^{-1}$.
**TGA**: the TGA data showed constant decline instead of a pronounced weight loss step, hence unambiguous quantification was not possible. A possible reason is the thermal instability of the aromatic nitrile and associated mass loss during decomposition.
**Kaiser test**: negative

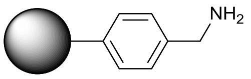


**7**

80 mg of diamond **6** was sonicated in 4 ml of $BH_3{\cdot}THF$ (1M) under nitrogen atmosphere for 10 min and afterwards the mixture was refluxed for 14 h. After cooling to room temperature excess borane was decomposed by adding 2 N HCl. The diamond particles were isolated by centrifugation. The precipitate was washed repeatedly five times with water, four times with acetone and two times with dichloromethane in consecutive washing / centrifugation cycles. 65 mg (81 % yield) of a gray solid was obtained as the product.

**Elemental analysis:** C: 89.1 %, H: 1.2 %, N: 2.6 %.

**FT-IR** (vacuum cell): $\tilde{\upsilon}$ = 3254, 2926 (CH), 2878 (CH), 1724, 1667 (NH), 1630, 1606, 1444, 1377, 1302, 1213, 1153, 1111, 838 $cm^{-1}$.
**TGA** (% weight loss): 160-500 °C: 2.0 % (fragment: $C_7H_8N$; 106 g $mol^{-1}$).
**Quant. Kaiser test:** UV: A = 0.6169 (566 nm).
**Surface loading:** 0.22 mmol $g^{-1}$ (calculated from EA)
0.19 mmol $g^{-1}$ (calculated from TGA).
0.17 mmol $g^{-1}$ (Kaiser-Test)

**Reaction of 2 with MCPBA (Control experiment for the side reaction during the synthesis of 10)**

25.0 mg of the annealed nanodiamond **2** was dispersed in a solution of 85.0 mg (0.49 mmol) of MCPBA in 5 ml dry dichloromethane and stirred for 20 h at room temperature under a nitrogen atmosphere. The nanodiamond was isolated by centrifugation and the supernatant discarded. The precipitate was washed six times with dichloromethane, six times with acetone and five times with water in consecutive washing / centrifugation cycles. 17 mg (68 % yield) of a gray solid was obtained as the product after drying at 70 °C for 24 h.
**Elemental analysis:** C: 88.5 %, H: 1.2 %, N: 2.4 %; **FT-IR** (vacuum cell): $\tilde{\upsilon}$ = 3410 (OH), 2925 (CH), 2854 (CH), 1721 (C=O), 1630 (OH), 1252, 1213, 1104, 807 $cm^{-1}$; **TGA** (% weight loss): 130-400 °C: 1.4 %.

## Additional analytical data of functionalized nanodiamond samples.

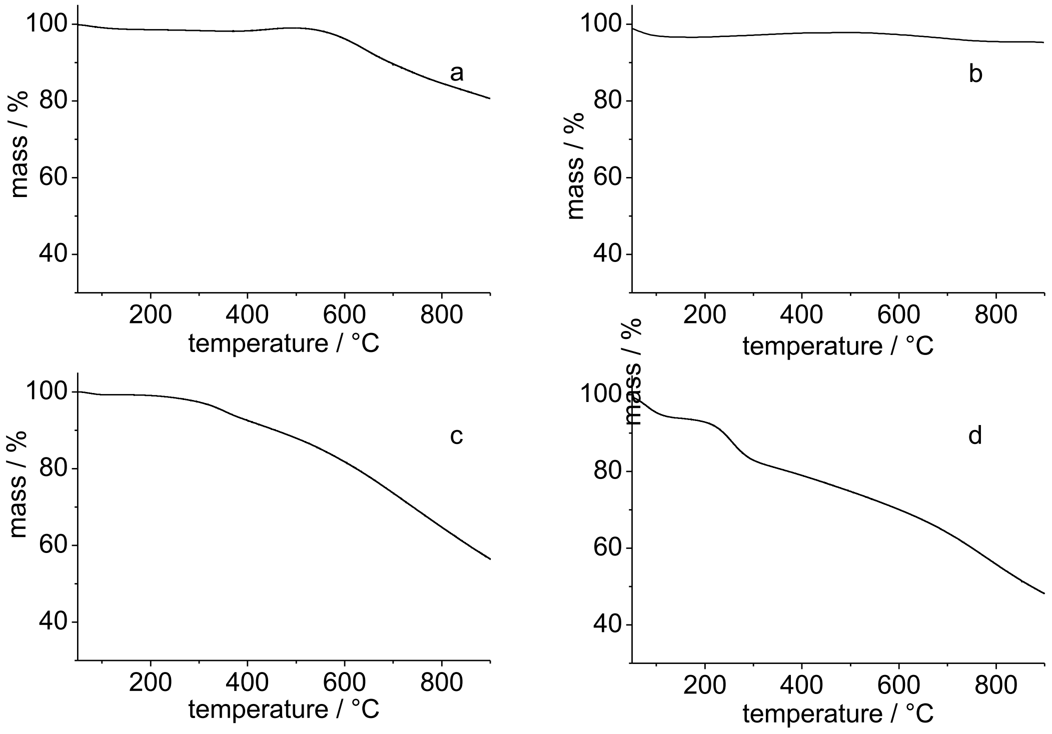


**Fig. 1S** Thermograms of pristine nanodiamond **1** (a), annealed nanodiamond **2** (b), nanodiamond **4** (c) and aminated nanodiamond **5** (d).

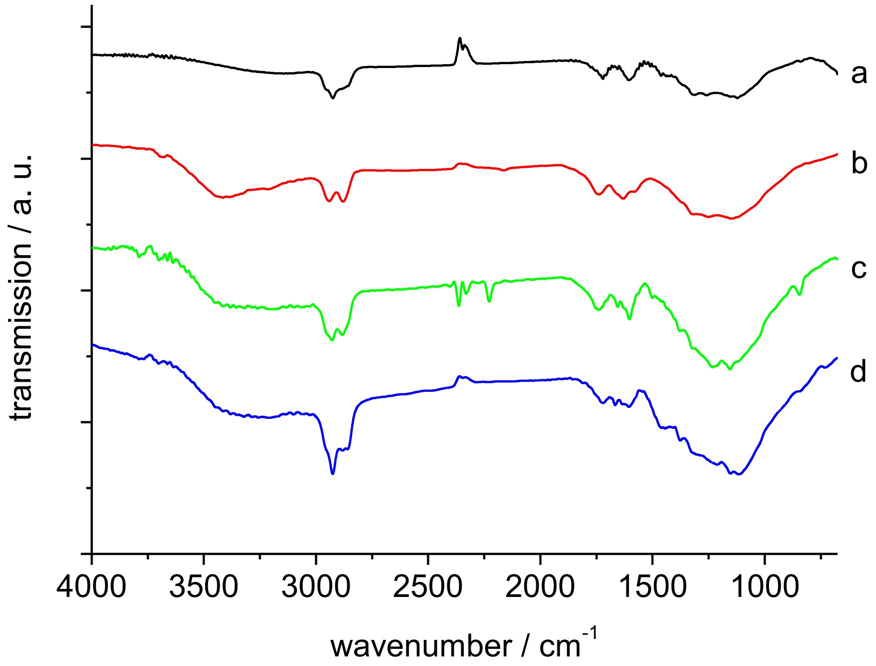


**Fig. 2S** IR-spectra of pristine nanodiamond **1** (a), annealed nanodiamond **2** (b), nitrile functionalized nanodiamond **6** (c) and aminated nanodiamond **7** (d). The inverted peaks around 2200-2300 $cm^{-1}$ are due to carbon dioxide from ambient air.

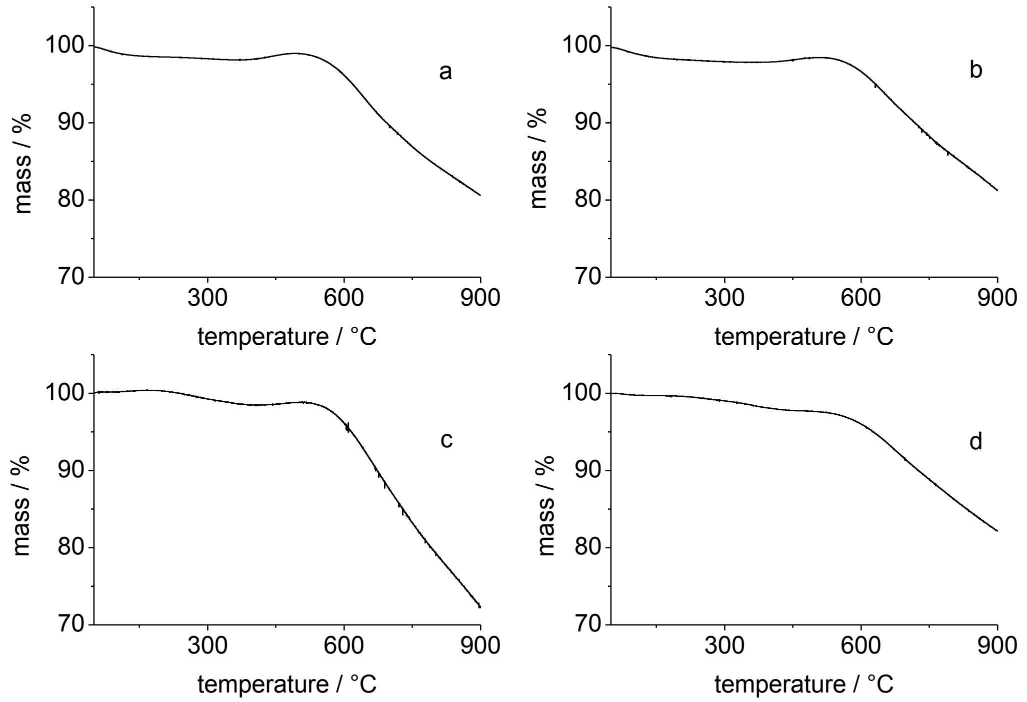


**Fig. 3S** Thermogram of pristine nanodiamond **1** (a), annealed nanodiamond **2** (b), nanodiamond **6** (c) and nanodiamond **7** (d).

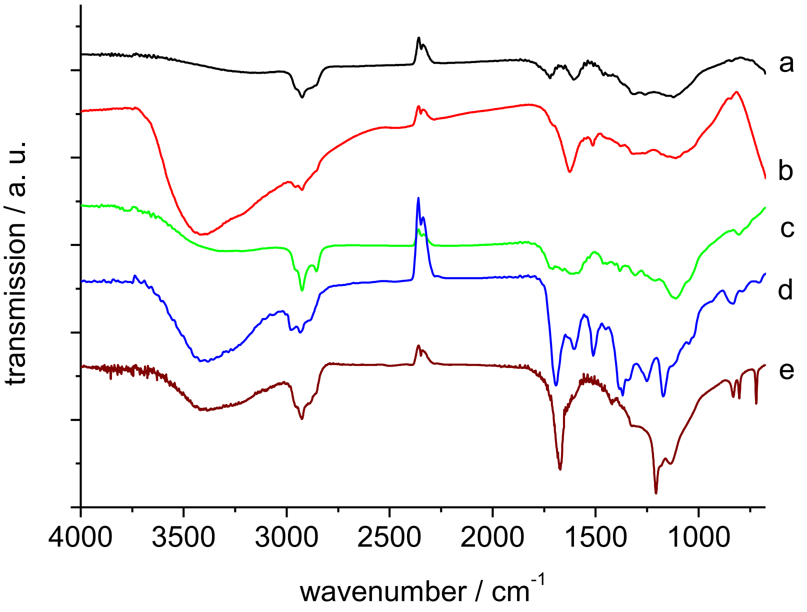


**Fig. 4S** IR-spectra of pristine nanodiamond **1** (a), hydroxylated nanodiamond **12** (b), silanized nanodiamond **13** (c), Boc-protected nanodiamond **14** (d) and aminated nanodiamond **15** (e). The inverted peaks around 2200-2300 $cm^{-1}$ are due to carbon dioxide from ambient air. The spectra are not normalized and are shown as obtained. The very strong OH band in spectrum (b) is due to the water adsorption on hydroxlyated nanodiamond **12** (which still was present after thorough vacuum drying of the pellet for IR measurement). In comparison samples **13-15** contain less water due their increased hydrophobicity caused by the organic moieties.

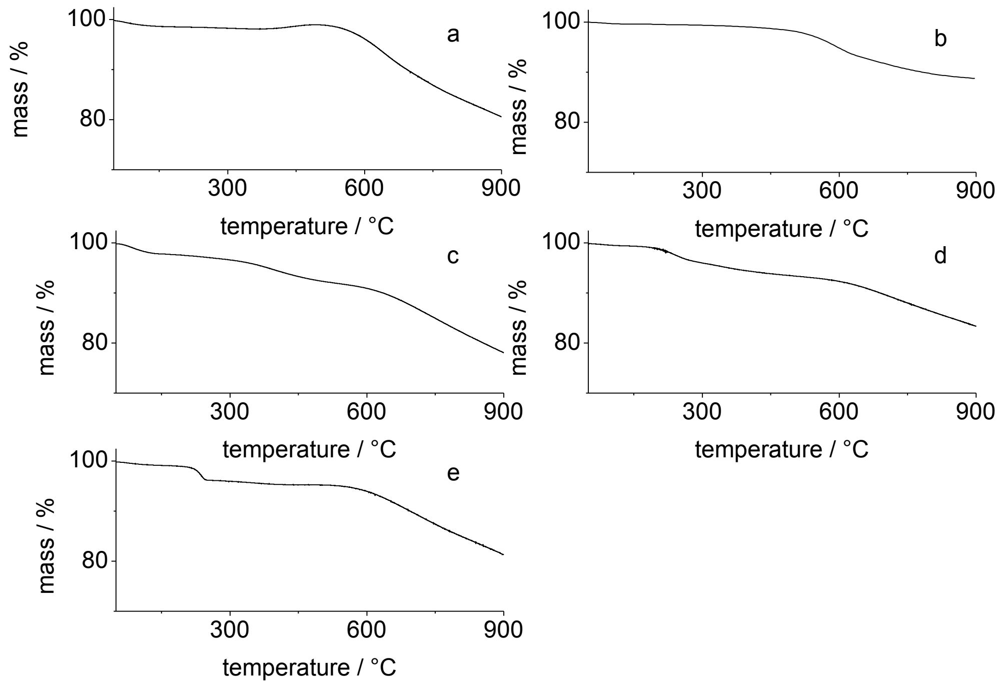


**Fig 5S** Thermogram of pristine nanodiamond **1** (a), hydroxylated nanodiamond **12** (b), silanized nanodiamond **13** (c), nanodiamond **14** (d) and nanodiamond **15** (e).

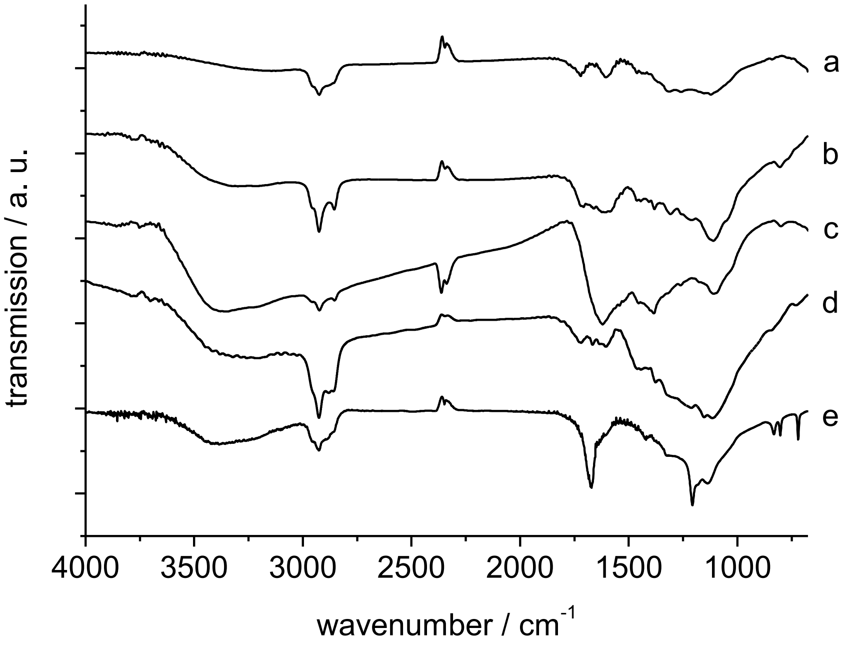


**Fig. 6S** Comparison of different aminated nanodiamond samples: FTIR-spectra of pristine nanodiamond **1** (a), aminosilanized nanodiamond **13** (b), pyrazine derived conjugate **5** (c), benzylamine functionalized nanodiamond **7** (d) and 4-aminophenylether functionalized nanodiamond **15** (e).

## Quantification of amino groups on nanodiamond using the modified Kaiser test

## Calibration of the photometric assay using benzylamine and optimization of the reaction time

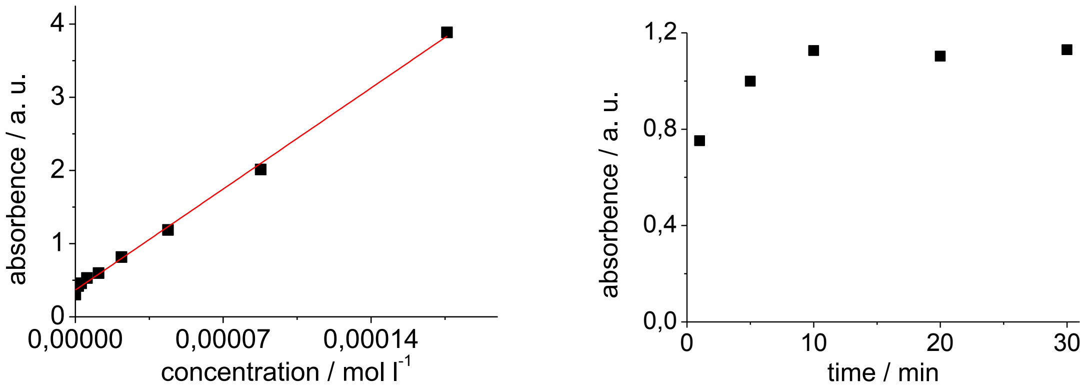


**Fig. 7S** Calibration curve with benzylamine (left) and reaction process over a period of 30 min. As can be seen from the duration graph at least 15 min (better 30 min.) should be applied as reaction time.

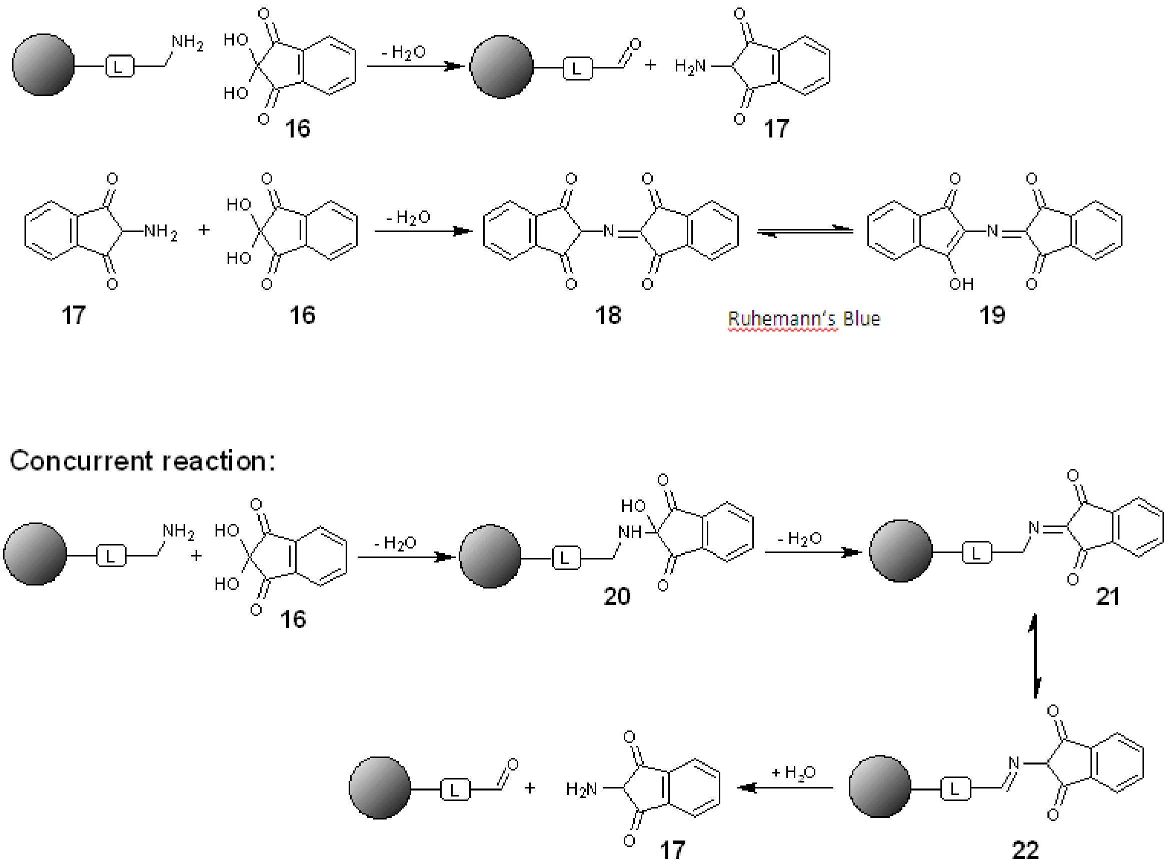


**Fig. 8S:** Proposed mechanism for the Kaiser test reactions on amino functionalized nanodiamond (top). A concurrent reaction is annihilating the required compound **16** if no precautions in the form of an increased amount of KCN and extended reaction time is taken.

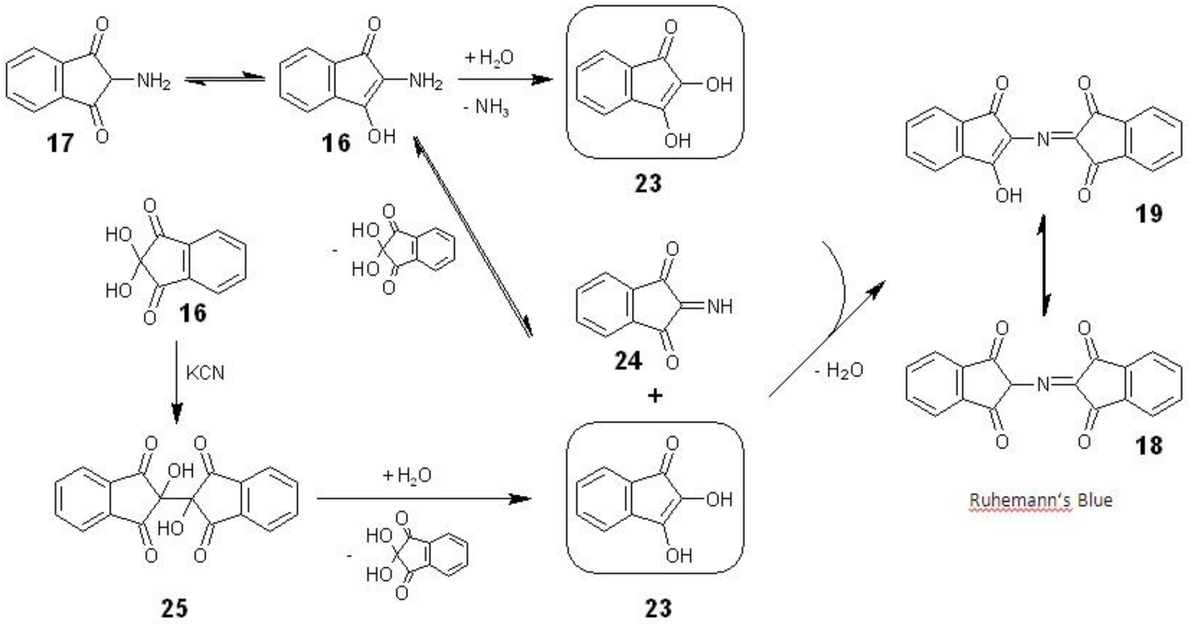


**Fig. 9S:** Reaction mechanism for the formation of Ruhemann's Blue.

**References:**


[1] A. Krueger, Y. Liang, G. Jarre, J. Stegk, *J. Mater. Chem.* **2006**, *16*, 2322-2328.

[2] Jaung, Jae-Yun; Matsuoka, Masaru; Fukunishi, Koushi, *Dyes and Pigments* **1997**, *34*, 255-266.

[3] M. D. Wendt, T. W. Rockway, A. Geyer, W. McClellan, M. Weitzberg, X. Zhao, R. Maitei, V. L. Nienaber, K. Stewart, V. Klinghofer, V. L. Giranda, *J. Med. Chem.* **2004**, *47*, 303-324.
